# Serum levels of NLRP3 and HMGB-1 are associated with the prognosis of patients with severe blunt abdominal trauma

**DOI:** 10.6061/clinics/2019/e729

**Published:** 2019-08-06

**Authors:** Kuanxue Sun, Hongwei Xia

**Affiliations:** I Department of General Surgery, GongLi Hospital of Shanghai Pu Dong New District, Shanghai, 200135, China; II Department of Ultrasound, GongLi Hospital of Shanghai Pu Dong New District, Shanghai, 200135, China

**Keywords:** NLRP3, HMGB-1, Severe Blunt Abdominal Trauma

## Abstract

**OBJECTIVES:**

To investigate the relationship between the serum levels of NLRP3 and HMGB-1 and the prognosis of patients with severe blunt abdominal trauma.

**METHODS:**

In total, 299 patients were included in the current study from July 2014 to December 2015. All patients were divided into the mild/moderate blunt abdominal trauma group and the severe blunt abdominal trauma group according to their injury severity scores. Serum levels of NLRP3 and HMGB-1 were measured upon admission (0 h) and at 12 h, 24 h, 48 h, 72 h and 7 days after admission.

**RESULTS:**

Compared with the healthy controls, both the mild/moderate and severe blunt abdominal trauma groups had higher serum levels of NLRP3 and HMGB-1 at admission. At all points, the serum levels of NLRP3 and HMGB-1 were significantly higher in the severe group than in the mild/moderate group. The serum levels of both NLRP3 and HMGB-1 were significantly higher in the deceased patients than in the living patients. The Kaplan-Meier curve showed that compared with patients with higher levels of NLRP3 or HMGB-1, those with lower levels had longer survival times. The serum levels of both NLRP3 and HMGB-1 were independent risk factors for 6-month mortality in severe blunt abdominal trauma patients.

**CONCLUSION:**

The serum levels of NLRP3 and HMGB-1 were significantly elevated in severe blunt abdominal trauma patients, and the serum levels of both NLRP3 and HMGB-1 were correlated with 6-month mortality in severe blunt abdominal trauma patients.

## INTRODUCTION

Trauma is the leading cause of death among people younger than 45 years, and it is also the fourth overall cause of death regardless of age; therefore, trauma imposes a substantial economic burden on health care systems 1,2. More than 180,000 people died of trauma in 2007, and almost one-third of all emergency department visits are due to trauma every year 3. Among all types of trauma, abdominal trauma accounts for a large proportion of trauma cases 4,5.

The most common causes of blunt abdominal trauma (BAT) are traffic accidents, falls from a height, assaults, and sports accidents 6,7. In the treatment of BAT, any delay in the identification of intra-abdominal injuries (IAI) may lead to morbidity and mortality 8. Thus, a timely and accurate diagnosis is always critical and may help promptly select an appropriate treatment strategy. There are many methods of diagnosing trauma and injury severity, such as exploratory laparotomy 9 and imaging methods, such as computed tomography (CT) scans 10. In recent years, the clinical significance of biomarkers for the diagnosis and prediction of the prognosis of trauma patients has been widely accepted 11. Many biomarkers, such as IL-1β 12 and TNF-α 13, are related to posttraumatic complications, such as systematic inflammation and sepsis 14.

High mobility group box-1 (HMGB-1), a member of the alarmin group of cellular messaging proteins, is a pro-inflammatory cytokine that has been proven to be associated with posttraumatic inflammation 15,16. Studies have demonstrated that NACHT domain-, leucine-rich repeat-, and PYD-containing protein 3 (NLRP3) plays an important role in HMGB-1-mediated inflammation in many diseases and bioprocesses. NLRP3 can facilitate *in vivo* HMGB-1 release 17, and HMGB-1 can induce an increase in the level of IL-1β by activating the NLRP3 inflammasome 18. However, to date, no study has focused on the role of NLRP3 in BAT, and few studies have reported the relationship between HMGB-1 and the prognosis of BAT patients.

The present study aimed to investigate the relationship between serum levels of NLRP3 and HMGB-1 and the prognosis of patients with severe BAT. This study investigated the role of NLRP3 and HMGB-1 in severe BAT and provides some new treatment targets for the treatment of severe BAT patients.

## MATERIALS AND METHODS

### Patients

In this study, 299 patients were included. All patients were diagnosed with BAT from July 2014 to December 2015 in our hospital. All patients were admitted to our hospital within 12h after experiencing BAT, and the diagnosis of BAT was confirmed by imaging methods such as CT scans. Patients were divided into groups according to their different injury severity scores (ISSs); the mild/moderate BAT group was defined as patients with ISSs<16, and the severe BAT group was defined as patients with ISSs≥16. All patients were between 18 and 60 years old. Patients with the following diseases or symptoms were excluded: patients who had severe infections before BAT; patients with severe systemic diseases such as severe cardiac, brain, liver, and renal diseases; and pregnant patients. All patients were treated according to the local management protocols for abdominal trauma. In addition, 50 healthy individuals were enrolled as the healthy controls. Written informed consent was obtained from all participants or their families within 24 h of admission. The present study was approved by the ethics committee of GongLi Hospital of Shanghai Pu Dong New District.

### Measurement and data collection

Data including age, sex, causes of injury and ISSs were recorded. Blood samples were collected upon admission (0 h) and at 12 h, 24 h, 48 h, 72 h and 7 days after admission. The serum levels of NLRP3 and HMGB-1 were both determined by enzyme-linked immunosorbent assay (ELISA) using commercially available NLRP3 and HMGB-1 ELISA kits (the HMGB-1 kit was purchased from Shino-Test Corporation, Kanagawa, Japan, and the NLRP3 kit was purchased from MyBioSource, San Diego, CA) according to the manufacturer's instructions. For the survival analysis, all-cause deaths were recorded for patients during hospitalization, and the follow-up period lasted for 6 months from the time of admission.

### Statistical analysis

The measurement data are expressed as the mean±SD. The chi-square test was used to compare the rates. Comparisons between two groups of continuous data were performed with Student's *t*-test, and comparisons among three or more groups were conducted using one-way analysis of variance (ANOVA) followed by Tukey’s post hoc test. Kaplan–Meier curves were generated for the survival analysis, and the relationship between the serum levels of NLRP3 and HMGB-1 and the 6-month mortality rate of patients was analyzed using a step-wise logistic regression model. A *p-*value less than 0.05 was considered statistically significant. All calculations were performed with SPSS 18.0.

## RESULTS

### Basic clinical information for all participants

The present study included 299 BAT patients. All patients were divided into the mild/moderate BAT group (n=135), which had a mean age of 38.15±11.65 years, a male:female ratio of 86:49, and a mean ISS of 11.78±1.76, and the severe BAT group (n=164), which had a mean age of 38.17±10.98 years, a male:female ratio of 106:58, and a mean ISS of 24.25±4.86. The ISS score of the mild/moderate BAT group was significantly lower than that of the severe BAT group (*p*<0.05) (Table 1). However, no significant differences were found with respect to age, gender or the causes of injury between the 2 groups. During the 6-month follow-up, 15 patients (9.15%) died in the severe BAT group, while all patients in the mild/moderate BAT group survived.

### Dynamic alterations in the levels of NLRP3 and HMGB-1 in BAT patients in different groups

**Figure 1:**
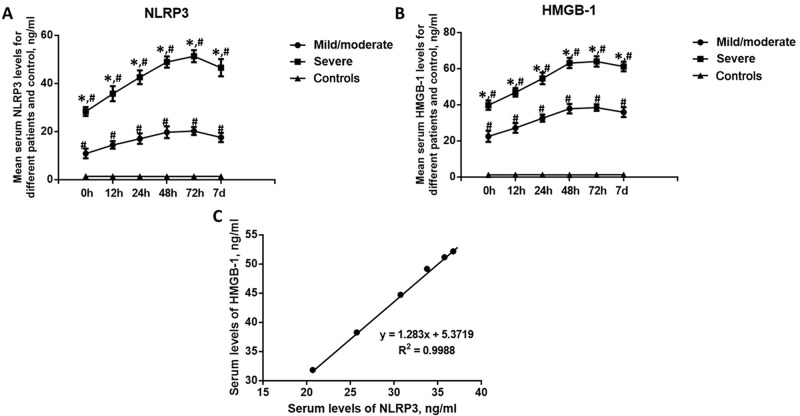
Dynamic changes in the serum levels of NLRP3 and HMGB-1 in BAT patients in different groups. A. Dynamic changes in the level of NLRP3; B. Dynamic changes in the level of HMGB-1; C. Correlation between the serum levels of NLRP3 and HMGB-1. *p<0.05, compared with the mild/moderate group; ^#^*p*<0.05, compared with the controls.

To study the roles played by NLRP3 and HMGB-1 in BAT patients, the dynamic alterations in the levels of NLRP3 and HMGB-1 in different groups of patients were determined at 0 h, 12 h, 24 h, 48 h, 7 2h and 7 days after admission. As shown in Figure 1, the serum levels of both NLRP3 and HMGB-1 were significantly higher in the mild/moderate and severe BAT groups of patients at admission than in the healthy controls (*p*<0.05). Meanwhile, at all time points, the serum levels of NLRP3 and HMGB-1 were significantly higher in the severe BAT group than in the mild/moderate BAT group (*p*<0.05). The serum levels of both NLRP3 and HMGB-1 increased at 72h in both patient groups compared to the levels at admission and then gradually returned to a normal level. In addition, the levels of NLRP3 and HMGB-1 in all patients at all points were found to be positively correlated with each other. These results indicated that the serum levels of NLRP3 and HMGB-1 might be associated with the effects of BAT.

### Dynamic alterations in the levels of NLRP3 and HMGB-1 in deceased and living patients with severe BAT

**Figure 2:**
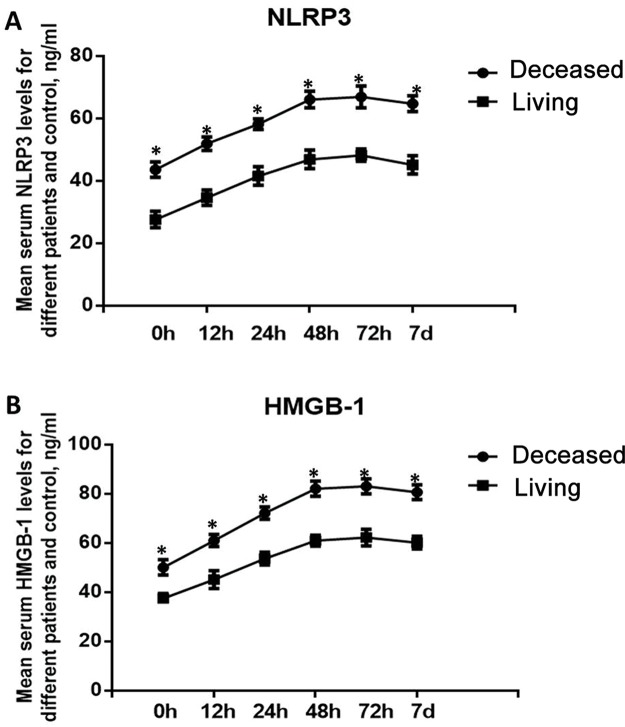
Dynamic changes in the serum levels of NLRP3 and HMGB-1 in deceased and living patients. A. Dynamic changes in the level of NLRP3; B. Dynamic changes in the level of HMGB-1. *p<0.05, compared with the living patients.

Then, we investigated the changes in the levels of NLRP3 and HMGB-1 in deceased and living patients in the severe BAT group. The results showed that at all time points, the serum levels of NLRP3 and HMGB-1 were significantly higher in the deceased patients than in the living patients (*p*<0.05), suggesting that NLRP3 and HMGB-1 levels might be associated with death in severe BAT patients (Figure 2).

### Correlations of NLRP3 and HMGB-1 with 6-month mortality in severe BAT patients

**Figure 3:**
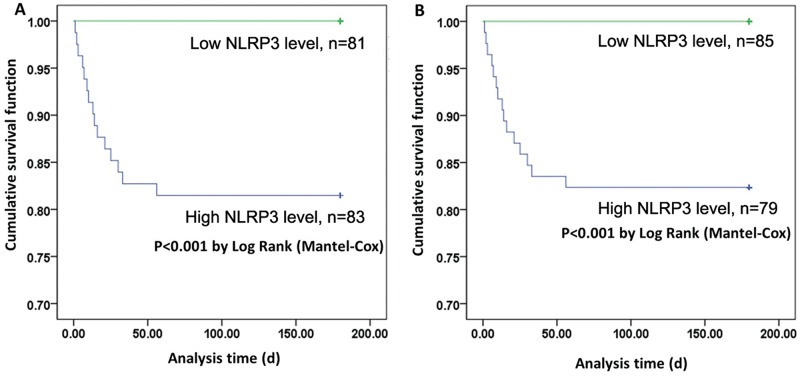
Kaplan–Meier curves for patients with high and low NLRP3/HMGB-1 levels.

Finally, we studied the correlation of serum NLRP3 and HMGB-1 levels with 6-month mortality in severe BAT patients (Table 2). Because rapid treatment is critical for patient survival, and the predictive significance of the serum levels of NLRP3 and HMGB-1 levels might be very helpful in diagnosing BAT, we investigated the NLRP3 and HMGB-1 levels at admission. Patients with severe BAT were divided into high or low NLRP3/HMGB-1 groups according to their mean serum NLRP3 and HMGB-1 concentrations. The Kaplan-Meier curve showed that patients with lower levels of NLRP3 or HMGB-1 had longer survival times than those of patients with higher levels of NLRP3 or HMGB-1 (*p*<0.05) (Figure 3). The subsequent logistic regression analysis also showed that the serum levels of both NLRP3 and HMGB-1 at admission were independent risk factors for 6-month mortality in severe BAT patients.

## DISCUSSION

It has been reported that traumatic injuries account for 9.6% of the global disease burden and are also the third most common cause of death in adolescents and adults ≤40 years 19,20. Among all types of trauma, abdominal trauma is one of the most important causes of morbidity and mortality. With regard to the treatment of trauma, prompt, accurate diagnoses are critical to determining the proper treatment strategy. In addition to traditional diagnostic methods, the clinical significance of many biomarkers for diagnosing and predicting the prognosis of numerous conditions has recently been studied. Among those biomarkers, HMGB-1 and NLRP3 are 2 newly identified factors that may be associated with the prognosis of trauma patients. Stahl et al. showed that patients with multiple severe traumas had significantly higher HMGB-1 levels than patients who had experienced moderate trauma or single fractures 21. An animal study by Liu et al. found that traumatic brain injury can induce the assembly of the NLRP3-inflammasome complex 22. Despite these studies, to the best of our knowledge, no study has focused on the roles played by NLRP3 and HMGB-1 in the prognosis of BAT patients.

In the present study, we first demonstrated that the serum levels of NLRP3 and HMGB-1 were significantly higher in all BAT patients than in the healthy controls, and the serum levels of NLRP3 and HMGB-1 were significantly higher in the severe BAT group than in the mild/moderate BAT group. Wallisch et al. studied the level of NLRP3 in the cerebrospinal fluid of severe traumatic brain injury patients and found that the level of NLRP3 was significantly increased in the cerebrospinal fluid after severe traumatic brain injury in infants and children and was also correlated with the ISSs of the patients 23. In an early study, Peltz et al. demonstrated that plasma HMGB1 levels were elevated within 1 h of injury in severe trauma patients more than 30-fold above the levels in healthy controls 24. All these results are consistent with our findings.

We also found that the levels of NLRP3 and HMGB-1 in all patients at all time points were positively correlated with each other. The relationship between NLRP3 and HMGB-1 has been investigated in many studies. Wei et al. showed that HMGB-1 promotes the activation of NLRP3 and caspase-8 inflammasomes via the NF-κB pathway in patients with acute glaucoma 25. Kim et al. found that HMGB-1 promotes IL-1β production in vascular smooth muscle cells through the activation of the NLRP3 inflammasome 18. However, until now, no clinical data have supported the relationship between NLRP3 and HMGB-1 in trauma patients.

Finally, we demonstrated that the serum levels of NLRP3 and HMGB-1 were correlated with 6-month mortality in severe BAT patients. Sousa et al. found that HMGB-1 levels were associated with shock within the first 72 h in patients after severe trauma 26. Zhang et al. demonstrated that NLRP3 polymorphisms were associated with the development of sepsis and multiple organ dysfunction syndrome in patients with major trauma 27. However, this is the first study to demonstrate a relationship between the serum levels of NLRP3 and HMGB-1 and mortality in trauma patients.

In conclusion, we conducted a prospective study to investigate the role of the serum levels of NLRP3 and HMGB-1 in the prognosis of patients with severe BAT. The results showed that the serum levels of NLRP3 and HMGB-1 were significantly elevated in severe BAT patients, and the serum levels of NLRP3 and HMGB-1 were correlated with 6-month mortality in severe BAT patients. This study was the first to demonstrate the roles played by NLRP3 and HMGB-1 in patients with severe BAT, providing new targets for the treatment of severe BAT patients.

## AUTHOR CONTRIBUTIONS

Xia H and Sun K participated in manuscript writing, analyzing the data and designing the study.

## Figures and Tables

**Table 1:** Basic clinical information of all participants.

Variable	Mild/moderate, n=135	Severe, n=164	Control, n=50
Mean age, years	38.15±11.65 (18-60)	38.17±10.98 (18-60)	38.49±10.99 (18-60)
Gender, male:female	86:49	106:58	32:18
Cause of injury, n (%)			
Traffic accident	73 (54.1%)	91 (55.5%)	
Fall	30 (22.2%)	35 (21.3%)	
Assault	23 (17.0%)	26 (15.9%)	
Other	9 (6.7%)	12 (7.3%)	
Mean ISS	11.78±1.76	24.25±4.86*	
Mortality during 6 months of follow-up, n (%)	0 (0%)	15 (9.15%)*	

* *p*<0.05, compared with the mild/moderate BAT group.

**Table 2:** Correlations between serum NLRP3/HMGB-1 levels and 6-month mortality in severe BAT patients according to the logistic multivariate regression analysis.

	Wald	Odds ratio	95% CI	*p-*value
NLRP3	11.925	2.842	(1.571∼5.140)	0.001
HMGB-1	10.576	3.919	(1.721∼8.925)	0.001
